# Genetic structure and triazole resistance among *Aspergillus fumigatus* populations from remote and undeveloped regions in Eastern Himalaya

**DOI:** 10.1128/msphere.00071-23

**Published:** 2023-06-21

**Authors:** Duanyong Zhou, Jianchuan Gong, Chengyan Duan, Jingrui He, Ying Zhang, Jianping Xu

**Affiliations:** 1 State Key Laboratory for Conservation and Utilization of Bio-Resources in Yunnan, Yunnan University, Kunming, China; 2 School of Life Science, Yunnan University, Kunming, China; 3 Key Laboratory of Biological Genetic Resources Mining and Molecular Breeding of Qianxinan Prefecture, Minzu Normal University of Xingyi, Xingyi, China; 4 Department of Biology, McMaster University, Hamilton, Ontario, Canada; University of Georgia, Athens, Georgia, USA

**Keywords:** aspergillosis, molecular markers, soil fungi, population genetics, antifungal resistance, geographic structure, allelic diversity, genotypic diversity

## Abstract

*Aspergillus fumigatus* is a ubiquitous mold and a common human fungal pathogen. Recent molecular population genetic and epidemiological analyses have revealed evidence for long-distance gene flow and high genetic diversity within most local populations of *A. fumigatus*. However, little is known about the impact of regional landscape factors in shaping the population diversity patterns of this species. Here we sampled extensively and investigated the population structure of *A. fumigatus* from soils in the Three Parallel Rivers (TPR) region in Eastern Himalaya. This region is remote, undeveloped and sparsely populated, bordered by glaciated peaks more than 6,000 m above sea level, and contained three rivers separated by tall mountains over very short horizontal distances. A total of 358 *A*. *fumigatus* strains from 19 sites along the three rivers were isolated and analyzed at nine loci containing short tandem repeats. Our analyses revealed that mountain barriers, elevation differences, and drainage systems all contributed low but statistically significant genetic variations to the total *A. fumigatus* population in this region. We found abundant novel alleles and genotypes in the TPR population of *A. fumigatus* and significant genetic differentiation between this population and those from other parts of Yunnan and the globe. Surprisingly, despite limited human presence in this region, about 7% of the *A. fumigatus* isolates were resistant to at least one of the two medical triazoles commonly used for treating aspergillosis. Our results call for greater surveillance of this and other human fungal pathogens in the environment.

**IMPORTANCE:**

The extreme habitat fragmentation and substantial environmental heterogeneity in the TPR region have long known to contribute to geographically shaped genetic structure and local adaptation in several plant and animal species. However, there have been limited studies of fungi in this region. *Aspergillus fumigatus* is a ubiquitous pathogen capable of long-distance dispersal and growth in diverse environments. In this study, using *A. fumigatus* as a model, we investigated how localized landscape features contribute to genetic variations in fungal populations. Our results revealed that elevation and drainage isolation rather than direct physical distances significantly impacted genetic exchange and diversity among the local *A. fumigatus* populations. Interestingly, within each local population, we found high allelic and genotypic diversities, and with evidence ~7% of all isolates being resistant to two medical triazoles, itraconazole and voriconazole. Given the high frequency of ARAF found in mostly natural soils of sparsely populated sites in the TPR region, close monitoring of their dynamics in nature and their effects on human health is needed.

## INTRODUCTION

*Aspergillus fumigatus* is the most predominant *Aspergillus* human-pathogenic species and the primary cause of aspergillosis (1). Two factors have contributed to its broad distributions: its ability to grow rapidly on a diversity of organic debris and producing very large numbers of airborne spores (2). In addition, the increasing emergence of azole resistance of *A. fumigatus* in both clinical and environmental settings limit the clinical efficacy of azoles, and subsequently increases the difficulty of the prevention and control of invasive aspergillosis (IA) (3 - 5). While healthy humans can typically control *Aspergillus* infection, the high dispersibility, global distribution, and an increase in drug resistance have led to increasing incidences of IA (6, 7). With increasing anthropogenic activities such as human travel and trade across the world, the occurrence of novel and introductions of genotypes with significant traits concerning drug resistance or virulence beyond their natural range are expected to increase (8, 9). Therefore, understanding the processes and natural ecological factors involved in the dispersal of the fungus is critical to reducing the threat of treatment failure and to managing aspergillosis outbreaks (10).

Due to their strong ability to cope with environmental changes, fungal spores represent a major force for dispersal and local adaptation in fungi (11). It has been suggested that free-living microbial eukaryotes smaller than 1–3 µM are highly mobile and can be effectively dispersed by wind over long distances; thus, blurring the potential effects of geographic separation on their genetic relationships (12, 13). Though spores (e.g., asexual spores, ascospores, and basidiospores) of many fungi are in that size range and found cosmopolitan, an increasing number of fungal species showed significant contributions of geography, topography, climate oscillations, ecological, and physical barriers to their genetic variation, such as in *Aspergillus* (9, 14), *Cryptococcus* (15, 16), *Neurospora* (17, 18), yeast (19, 20), etc. In *A. fumigatus*, the effects on prominent local and regional landscape factors, such as steep mountains and deep gorges to genetic variations remain to be investigated. Inferring the driving forces for the genetic structure of fungi has been one of the central topics in population genetics. It is of vital importance to understand the demographic history of endemic populations, the origins and spread of novel genotypes, the epidemiology of fungal diseases, and spread of drug resistance (21). The development and application of a diversity of molecular markers over the last 30 years have greatly enhanced our ability to track fungal population history and dynamics (22, 23).

The Himalaya–Hengduan Mountains region is in Southwest China and has many spectacular topographic features triggered by the rapid uplifting of the Qinghai–Tibetan Plateau (QTP) and the Quaternary climate oscillations. It’s also one of the global biodiversity hotspots (24). The patterns of genetic diversity and genetic structures of plants and animals in the QTP and its adjacent regions have attracted broad attention from researchers for several decades (25, 26). Over 4,000 fungal species, representing about 40% of known fungal taxa in China, have been reported in this area (27, 28). However, the relationships of genetic structure with the geological and climate changes, as well as the evolutionary histories of fungi, are relatively poorly understood in this region. Significantly, the main upstream water of three great rivers of Asia: the Yangtze (Jinsha River), Mekong (Lancang River), and Salween (Nu River) run roughly parallel within this region through the steep gorges up to 3,000 m deep (29). Moreover, the Three Parallel Rivers (TPR) region is flanked by glaciated mountains of more than 6,000 m above sea level, featuring extreme changes in elevation over very short horizontal distances (30). The flanking peaks and the mountains separating the rivers in the TPR region constitutes both a natural barrier (mountains) for biological isolation and linear corridors (rivers) for the dispersal of genetic elements (e.g., fungal spores) (31). Here, we hypothesize that the geographical barriers, historical topographic events, and drainage isolation might limit the dispersal of the spores of *A. fumigatus* and thus have a significant influence on the genetic structure of *A. fumigatus*. Furthermore, we are interested in whether the deep and linear corridors along rivers provide easy passages for gene flow in this species.

Our previous studies revealed that greenhouse populations of *A. fumigatus* around metropolitan Kunming, the capital of Yunnan, had a high prevalence of azole-resistant *A. fumigatus* (ARAF) while a lower frequency of ARAF was identified in outdoor environments associated with fewer human activities (32). Thus, the use of azole fungicides for plant protection was suggested as a significant driver of the high-frequency ARAF in Yunnan (9). In addition, widespread recombination and genetic exchange within and among geographically and genetically divergent strains have been found, contributing to the high genetic diversity of *A. fumigatus* within local populations (9, 32). Furthermore, when compared to a global sample, we found abundant new alleles and new genotypes of *A. fumigatus* in Yunnan (9, 32). Together, these results suggested that the factors influencing *A. fumigatus* population structure are highly complex.

The highly heterogenous topographic and climatic diversities within the TPR region provide an excellent opportunity to investigate the potential geographic effects on *A. fumigatus* populations. In this study, we sampled extensively and investigated the population structure of *A. fumigatus* from the TPR region. A panel of nine short tandem repeats (STRs) markers, which have been widely used for genotyping strains of *A. fumigatus* (33), were used to determine the genotype of our strains. In addition, we investigated the susceptibilities of the strains to two common medical triazoles used for treating aspergillosis, itraconazole and voriconazole. For both triazole-susceptible and triazole-resistant strains, we analyzed the DNA sequences at the azole target gene *cyp51A*. The obtained genotype information was compared with those from other parts of Yunnan and from several other parts of the world. We aimed to: (i) reveal the genetic structure of the *A. fumigatus* in the TPR region, (ii) explore the relative roles of geographical barriers and landscape features in shaping the population genetic diversities of this species, and (iii) investigate the prevalence of triazole-resistant strains and their potential mutations associated with drug resistance.

## MATERIALS AND METHODS

### Soil samples, *A. fumigatus* isolation and identification

**FIG 1:**
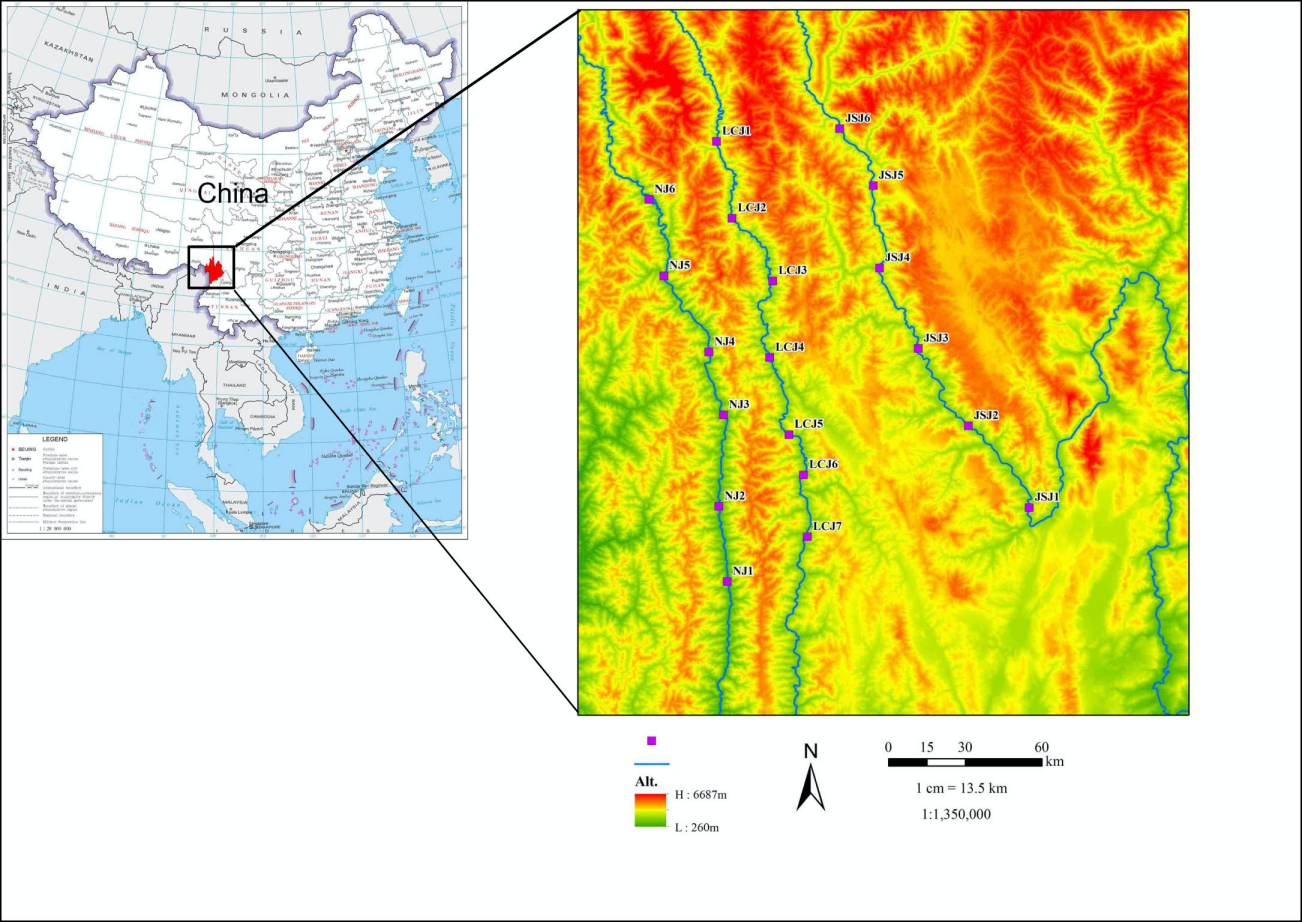
Geographical distribution of sampling sites.

A total of 1,900 soil samples were collected from 3 January to 7 January 2020 from the TPR region. There were 6–7 sampling sites along each of three rivers: Yangtze (Jinsha Jiang[JSJ]), Mekong (Lancang Jiang[LCJ]), and Salween (Nu Jiang[NJ]). Sampling sites belonging to the same river system were about 30 km apart from each other in numerical order. Along the three rivers, five sites along each river were located at similar altitudes as those from two other rivers (3 × 5 = 15 sites). The corresponding sites from different rivers were located very close to each other if measured based on map distance. At each site, 100 soil samples of about 10 g each were collected at a depth of 0–5 cm from the surface, then put into sterile zip-log plastic bags. Individual soil samples were about 2 m apart from each other. Together, the sampling sites covered about 21,323.25 km^2^ of the TPR’s core area. The detailed information about the sampling sites is shown in Table S1; Fig. 1. *A. fumigatus* isolation followed the method described previously (8). The initial and final identification followed the methods described in our previous study (9, 32).

### STR genotypes and population genetic analyses

All *A. fumigatus* isolates were genotyped by a panel of nine highly polymorphic STRs markers (STRAf 2A, 2B, 2C, 3A, 3B, 3C, 4A, 4B, and 4C), to understand the relationships among strains and populations of *A. fumigatus* from TPR. The number of tandem repeats at each locus for each strain was determined following the method described previously (33). The alleles at the nine STR loci were combined to generate the multilocus STR genotype for each strain. Furthermore, genotypic data of *A. fumigatus* isolates from our previous studies and from other countries reported previously and deposited in the STRAf database (http://afumid.shinyapps.io/afumID) were extracted and compared with *A. fumigatus* from TPR in this study (9, 14, 32).

*A. fumigatus* isolates from each of the 19 sampling sites were defined as belonging to one local geographic population in the population genetic analyses. Depending on the analyses, those individual local geographic populations were reassembled according to their river affiliation, altitude, longitude, and latitude similarity to investigate the relationships between genetic variation and various geographic parameters. We used GenAlEx v6.1 software to calculate the level of genetic differentiation among and within the 19 local geographical populations from the TPR area, and that among the TPR, other areas in Yunnan province, and the global samples (34). Mantel test was used to investigate the correlations between genetic and geographical distances among populations. Analysis of molecular variance (AMOVA) was used to estimate the relative contributions of geographic separation and different population subdivisions, as aforementioned, to the overall genetic variation. The program STRUCTURE v2.3.3 was performed to determine the optimal number (K) of genetic clusters from TPR and in the combined dataset that included all 750 global samples from our previous study (35). Principal coordinate analysis (PCoA) and discriminate analysis of principal components (DAPC) were used to investigate the genetic relationship between *A. fumigatus* isolates.

We constructed two minimum spanning trees (MSTs) based on STR markers (BIONUMERICS v8.0, Applied Maths, Belgium). One investigated the genetic relationships among isolates from TPR and those from our two previous studies including 233 strains from nine greenhouses in Jinning and 452 outdoor isolates from 19 geographic locations across Yunnan (9, 32). The second investigated the genetic relationships between isolates from TPR and a global sample (35). MEGA v6 was used to determine the genetic relationships between populations of *A. fumigatus* from TPR (36). IBM SPSS statistical software v22.0 was used to estimate the correlation between population diversity (Number of Effective Alleles, Shannon’s Information Index, Diversity and Unbiased Diversity) and longitude, latitude, and altitude, respectively. To examine the reproductive mode in natural populations of *A. fumigatus* from TPR, the index of association (IA), rBarD, and the proportion of pairwise loci that were phylogenetically compatible (PrC) were calculated following the method described previously (37).

### Susceptibility of *A. fumigatus* isolates and cyp51a gene sequencing

Two clinical azole drugs (ITR and VOR) commonly used for the treatment of aspergillosis were used to test the susceptibility of *A. fumigatus* isolated in this study following the methods described in the CLSI M38-A2 (38) and our previous studies (9, 32). Primer pairs A7 (5'-TCATATGTTGCTCAGCGG-3') and P450-A2 (5'-CTGTCTCACTTGGATGTG-3') (39) were used to amplify the *cyp51A* gene (including full-length coding and promoter regions) of all isolates obtained in this study.

## RESULTS

### Genotyping of *A. fumigatus* isolates from local populations

In this study, a total of 358 *A*. *fumigatus* strains were isolated from 1,900 soil samples from the 19 sites in the TPR core area. Two hundred fifteen alleles and 332 multilocus genotypes were identified across the nine STR loci. The number of alleles ranged from 10 to 48 per locus among the nine STR loci, with an average of 24. The STRAf3A locus had the most alleles (*n* = 48), followed by STRAf3C locus (*n* = 41). Among the total 215 alleles, 174 were shared between at least two of the sites from TPR. The remaining 41 alleles were found only in one site each. The 19 sampling sites from TPR differed in their total numbers of alleles, with the number of alleles ranging from 46 (JSJ5) to 99 (NJ5). Except three populations (JSJ5, LCJ2, and NJ4), private alleles were found in all other populations: 18, 14, and 9 private alleles were found in the Nu River, Jinsha River, and Lancang River, respectively (Table 1). Of the total 332 multilocus genotypes, only four were shared by two or more local populations and the remaining 328 were unique to individual local populations.

**TABLE 1:** Allelic information at nine STR loci for 19 local populations of *A. fumigatus* from the Three Parallel Rivers region in Yunnan, China

Population	No. of strains	No. of strains (clone-corrected)	No. of alleles (no. of private alleles)									
			2A	2B	2C	3A	3B	3C	4A	4B	4C	Total
JSJ1	22	17	5	6 (1)	9	13 (1)	9 (1)	12	7	6	4	71 (3)
JSJ2	22	22	6	7	7	17 (1)	11	13 (1)	6 (1)	5	4	76 (3)
JSJ3	18	18	5	6	5	15	10	14	5	4	5 (1)	69 (1)
JSJ4	22	21	8	10	7	17 (1)	10 (1)	16 (2)	6	5 (1)	4	83 (5)
JSJ5	11	11	4	4	5	6	8	7	4	6	2	46
JSJ6	12	12	5	5	7	11	11	8	8 (1)	5 (1)	4	64 (2)
LCJ1	22	21	4	6	6	14	11 (1)	13	8 (1)	4	4	70 (2)
LCJ2	13	12	7	3	6	10	10	7	4	3	3	53
LCJ3	21	20	3	5	7	16	11	15	7 (1)	3	2	69 (1)
LCJ4	22	22	3	4	5	16	9	14 (1)	4	3	3	61 (1)
LCJ5	19	17	5	6	6	13	10	12 (1)	4	5 (1)	2	63 (2)
LCJ6	22	21	5	8	7	11	13 (1)	10	4	3	2	63 (1)
LCJ7	22	22	7	7	9	16	12 (1)	15 (1)	9	4	3	82 (2)
NJ1	22	19	8	7	11 (2)	14 (1)	9	15	8	5 (1)	4	81 (4)
NJ2	13	13	5	8	9 (1)	10	9	11	6	4	3	65 (1)
NJ3	12	12	5	5	6	10 (1)	7	10	5	4	4	56 (1)
NJ4	18	14	6	8	8	10	9	11	8	5	4	69
NJ5	21	21	10	11 (1)	13 (1)	19 (1)	15	17 (1)	7	4	3	99 (4)
NJ6	24	21	8 (1)	7 (1)	12 (1)	16	12 (3)	10	6	6	6 (2)	83 (8)
Total	358	336	16	19	22	48	29	41	18	12	10	215 (40)

The number of effective alleles (Ne) from the 19 local populations from TPR ranged from 3.401 (JSJ5) to 8.637 (NJ5), with an average of 5.455. Shannon’s Information Index range from 1.288 (JSJ5 population) to 2.094 (NJ5 population), with an average of 1.643. Simpson’s Diversity ranged from 0.563 (LCJ4 population) to 0.821 (NJ5 population), with an average of 0.705. Unbiased Diversity range from 0.594 (LCJ4 population) to 0.863 (NJ5 population), with an average of 0.751 (Table 2). When taking local populations along the same river as one meta-population, the genetic diversity of *A. fumigatus* in the Nu River population was the highest (uh = 0.838), followed by the Jinsha River population (uh = 0.754), and the Lancang River population (uh = 0.705). However, analysis of variance using the mean diversity estimates of each river showed no significant difference in the pairwise comparisons among the three rivers (Table S2).

**TABLE 2:** Allelic diversities within the 19 local populations of *A. fumigatus* from the Three Parallel Rivers region in Yunnan, China

Sampling site	Effective alleles (Ne)	Shannon’s information index (I)	Simpson’s diversity (H)	Unbiased diversity (uh)
JSJ1	5.378	1.719	0.737	0.783
JSJ2	5.116	1.576	0.647	0.678
JSJ3	5.321	1.553	0.659	0.699
JSJ4	6.757	1.843	0.755	0.793
JSJ5	3.401	1.288	0.625	0.689
JSJ6	5.263	1.693	0.748	0.816
LCJ1	5.486	1.662	0.719	0.759
LCJ2	4.269	1.416	0.648	0.707
LCJ3	5.645	1.524	0.642	0.678
LCJ4	4.717	1.309	0.563	0.590
LCJ5	4.900	1.499	0.654	0.696
LCJ6	3.874	1.409	0.621	0.653
LCJ7	6.084	1.784	0.740	0.775
NJ1	6.640	1.863	0.767	0.808
NJ2	5.620	1.712	0.750	0.812
NJ3	4.573	1.558	0.721	0.786
NJ4	5.492	1.797	0.785	0.845
NJ5	8.637	2.094	0.821	0.863
NJ6	6.464	1.919	0.798	0.838
Mean	5.455	1.643	0.705	0.751

### Genetic variation within and among local populations in the Three Parallel Rivers region

AMOVA based on clone-corrected data showed that most genetic variation was found within local geographical populations. When combining the local populations according to river affiliations, upstream and downstream regions, and different altitude ranges, we found that except for the division of upstream and downstream rivers, all three levels contributed significantly to the overall genetic variation, with the regional level (among rivers, and among populations from different altitudes) contributing significantly to the overall genetic variations (3% and 2%, respectively, *P* = 0.01 for both, Table 3). Furthermore, there are significant genetic variances among sites within individual rivers, i.e., Jinsha River (PhiPT = 0.021, *P* = 0.01), Lancang River (PhiPT = 0.023, *P* = 0.001), and Nu River (PhiPT = 0.013, *P* = 0.004). We further investigated the extent of genetic differentiation between pairs of TPR populations. We found that the genetic differentiation level of the Nu River population was lower than that of the other two rivers (Table S4). In particular, except for one population from Jinsha River (JSJ6), the populations from the Nu River were significantly different from those along Lancang River and Jinsha River (*P* < 0.05) (Table S4). Among the 171 population pairs, 110 pairs showed statistically significant differentiation (*P* < 0.05). The biggest differentiation was found between LCJ4 population and NJ3 population (PhiPT = 0.141, *P* = 0.001), followed by that between LCJ4 population and NJ5 population (PhiPT = 0.122, *P* = 0.001). Similarly, significant genetic differentiation was also found between rivers, i.e., Jinsha River populations and Lancang River populations (PhiPT = 0.023, *P* = 0.001), Jinsha River populations and Nu River populations (PhiPT = 0.039, *P* = 0.001), and Lancang River populations and Nu River populations (PhiPT = 0.044, *P* = 0.001) (Table S4).

**TABLE 3:** Summary results of AMOVA among regions of the *A. fumigatus* isolates from different sources

Source	df	SS	MS	Est. Var.	%	Value	*P*
River isolations							
Among rivers	2	33.477	16.739	0.11	3%	0.031	0.01
Among sites	16	72.541	4.534	0.066	2%	0.019	0.01
Within sites	317	1,072.75	3.384	3.384	95%	0.049	0.01
Total	335	1,178.768		3.559	100%		
Upstream and downstream divisions							
Among river sections	4	19.558	4.89	0	0%	−0.01	1
Among sites within sections	10	64.355	6.436	0.182	5%	0.052	0.01
Within sites	246	819.741	3.332	3.332	95%	0.043	0.01
Total	260	903.655		3.514	100%		
Altitudinal differences							
Among altitudinal ranges	5	44.179	8.836	0.078	2%	0.022	0.01
Among sites within altitudinal groups	13	61.839	4.757	0.077	2%	0.022	0.01
Within sites	317	1,072.75	3.384	3.384	96%	0.044	0.01
Total	335	1,178.768		3.539	100%		
Populations along Jinsha River							
Among sites along Jinsha River	5	22.586	4.517	0.071	2%	0.021	0.015
Within sites	95	317.236	3.339	3.339	98%	0.021	0.015
Total	100	339.822		3.41	100%		
Populations along Lancang River							
Among sites along Lancang River	6	27.198	4.533	0.073	2%	0.023	0.001
Within sites	127	400.004	3.15	3.15	98%	0.023	0.001
Total	133	427.201		3.222	100%		
Populations along Nu River							
Among sites along Nu River	5	22.757	4.551	0.049	1%	0.013	0.004
Within sites	95	355.51	3.742	3.742	99%	0.013	0.004
Total	100	378.267		3.791	100%		
Globe							
Among geographic pops	11	295.934	26.903	0.392	9%	0.095	0.001
Within pops	997	3,725.489	3.737	3.737	91%	0.095	0.001
Total	1,008	4,021.423		4.128	100%		

When combining local populations with similar altitudes as one meta-population, the diversity of *A. fumigatus* was higher in regions among altitudes 1360–1450 m (uh = 0.860), followed by 1160–1290 m (uh = 0.803) and the lowest in 2740 m and higher region (uh = 0.689). When combining local populations scattered in similar latitude ranges as one meta-population, we found that the diversity of *A. fumigatus* was highest in population group in middle latitudes (ranging from 27.73 to 27.78 N) (uh = 0.804), followed by the group in high latitude area (ranging from 27.95 to 28.07 N) (uh = 0.792), and the lowest genetic diversity was found in population group with lowest latitude (ranging from 27.46 to 27.49 N) (uh = 0.722) (Table S3). However, unlike those based on allele-frequencies, none of the pairwise genetic diversity index comparisons yielded statistically significant differences.

### Relationship between genetic distance and geographical parameters

**FIG 2:**
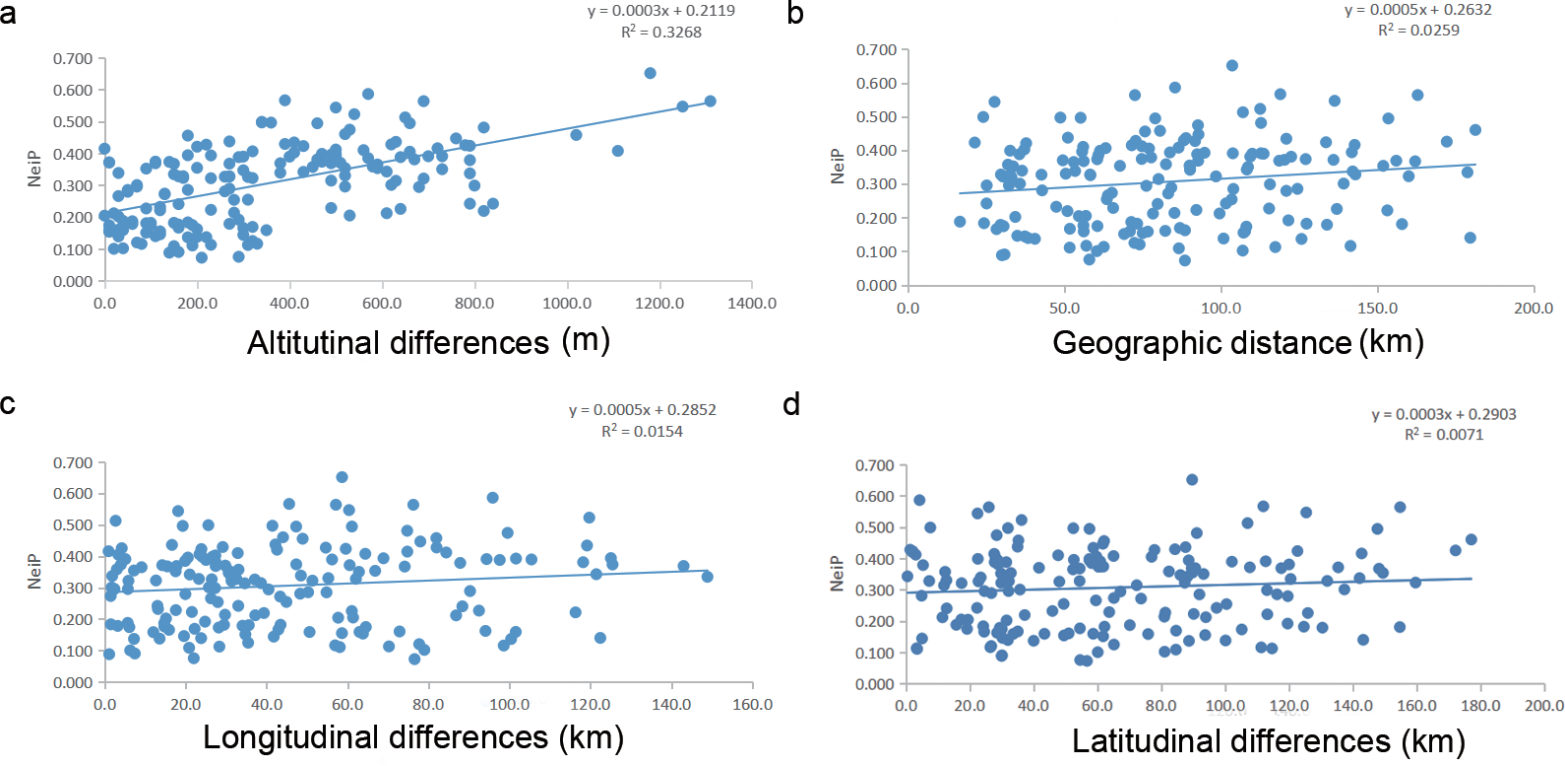
Results from four Mantel tests between genetic differences and altitudinal differences (a), the two-dimensional geographical distances (based on longitudinal and latitudinal coordinates) (b), longitudinal differences (c) and latitudinal differences (d) between populations.

We further quantified the relationship between genetic variation and geographical parameters. Specifically, the observed differences between samples separated by altitudes showed a significant positive correlation with the genetic distances (*P* = 0.01) (Fig. 2a). In contrast, those of the geographical distances (*P* = 0.099) (Fig. 2b), longitudinal (*P* = 0.18) (Fig. 2c), and latitudinal (*P* = 0.24) (Fig. 2d) distance showed a weak and statistically insignificant positive correlation with the genetic distances. Interestingly, correlation analysis showed that altitude has a significant negative correlation with Shannon’s Information Index (R = −0.473, *P* = 0.041), Simpson’s Diversity (R = −0.488, *P* = 0.034), and Unbiased Diversity (R = −0.480, *P* = 0.038), respectively (Table 4).

**TABLE 4:** Correlation analysis between population genetic diversity indices with longitudinal, latitudinal, and altitudinal differences

Analyzed pairs of quantitative traits	R (correlation coefficient)	*P* value
The number of effective alleles (Ne) vs longitude	−0.309	0.198
The number of effective alleles (Ne) vs latitude	−0.032	0.895
The number of effective alleles (Ne) vs altitude	−0.409	0.082
Shannon’s information index (I) vs longitude	−0.294	0.222
Shannon’s information index (I) vs latitude	−0.067	0.784
Shannon’s information index (I) vs altitude	−0.473	0.041
Simpson’s diversity (h) vs longitude	−0.347	0.145
Simpson’s diversity (h) vs latitude	0.001	0.997
Simpson’s diversity (h) vs altitude	−0.488	0.034
Unbiased diversity (uh) vs longitude	−0.351	0.14
Unbiased diversity (uh) vs latitude	0.046	0.851
Unbiased diversity (uh) vs altitude	−0.48	0.038

### Population structure

**FIG 3:**
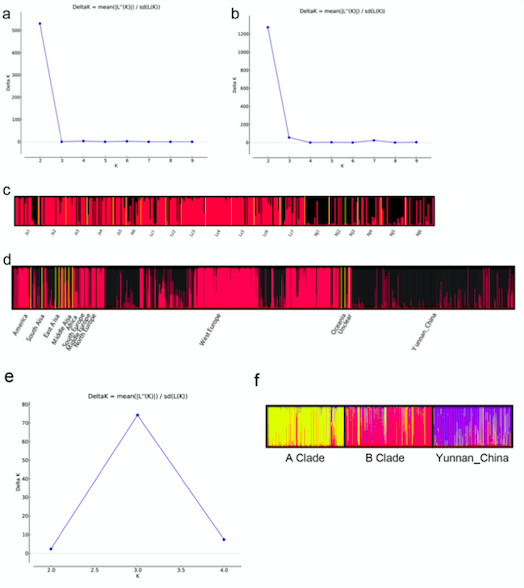
Genetic structuring results obtained from the STRUCTURE analysis. Plot of K against delta K (a) and analyses (c) for TPR populations. Plot of K against delta K (b) and analysis (d) using other geographical populations from Yunnan and around the world, plot of K against delta K (e) and analysis (f) using global A and B genetic populations and populations from Yunnan.

**FIG 4:**
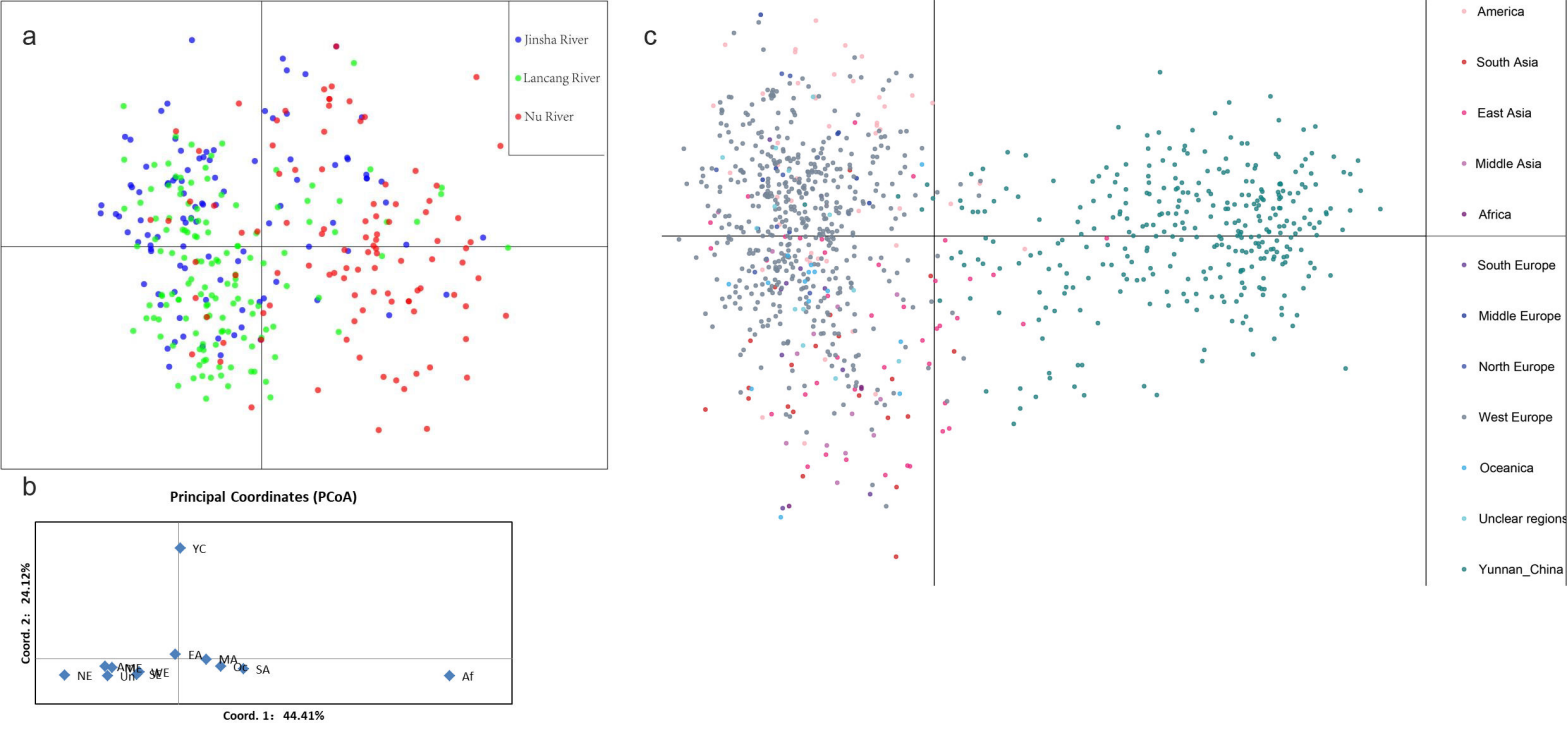
Results of DAPC analysis between TPR populations (a) and combined with global populations (c), and PCoA based on pairwise population genetic distances (b).

**FIG 5:**
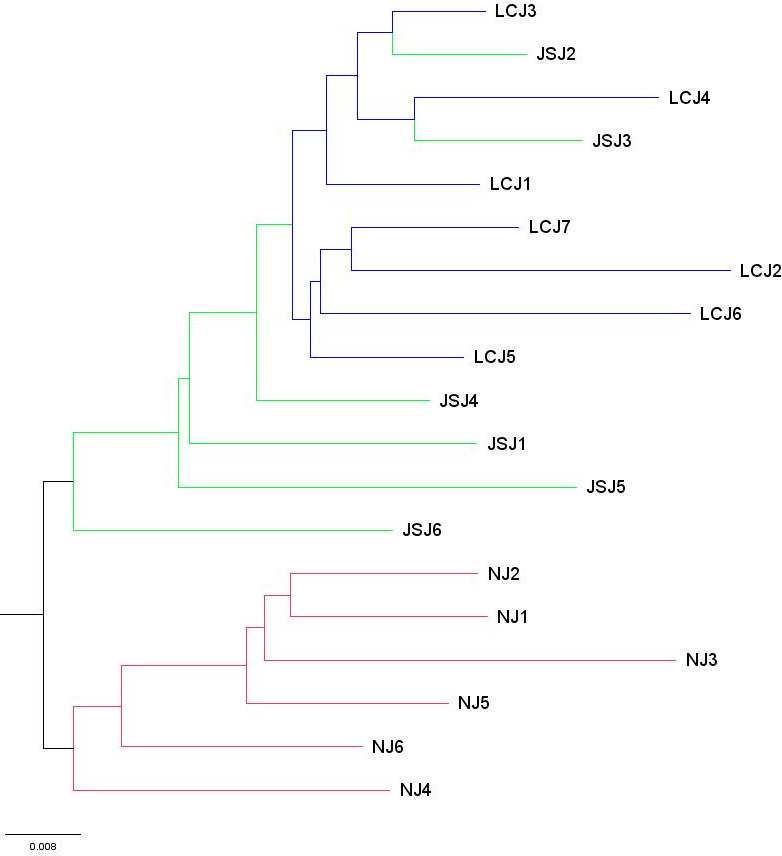
Genetic clustering of 19 *A*. *fumigatus* local populations from the TPR region based on 9 STR loci by Nei distance. Abbreviations: Am, America; SA, South Asia; EA, East Asia; MA, Middle Asia; Af, Africa; SE, South Europe; ME, Middle Europe; NE, North Europe; WE, West Europe; Oc, Oceanica; Un, Unclear regions; YC, Yunnan_China

**FIG 6:**
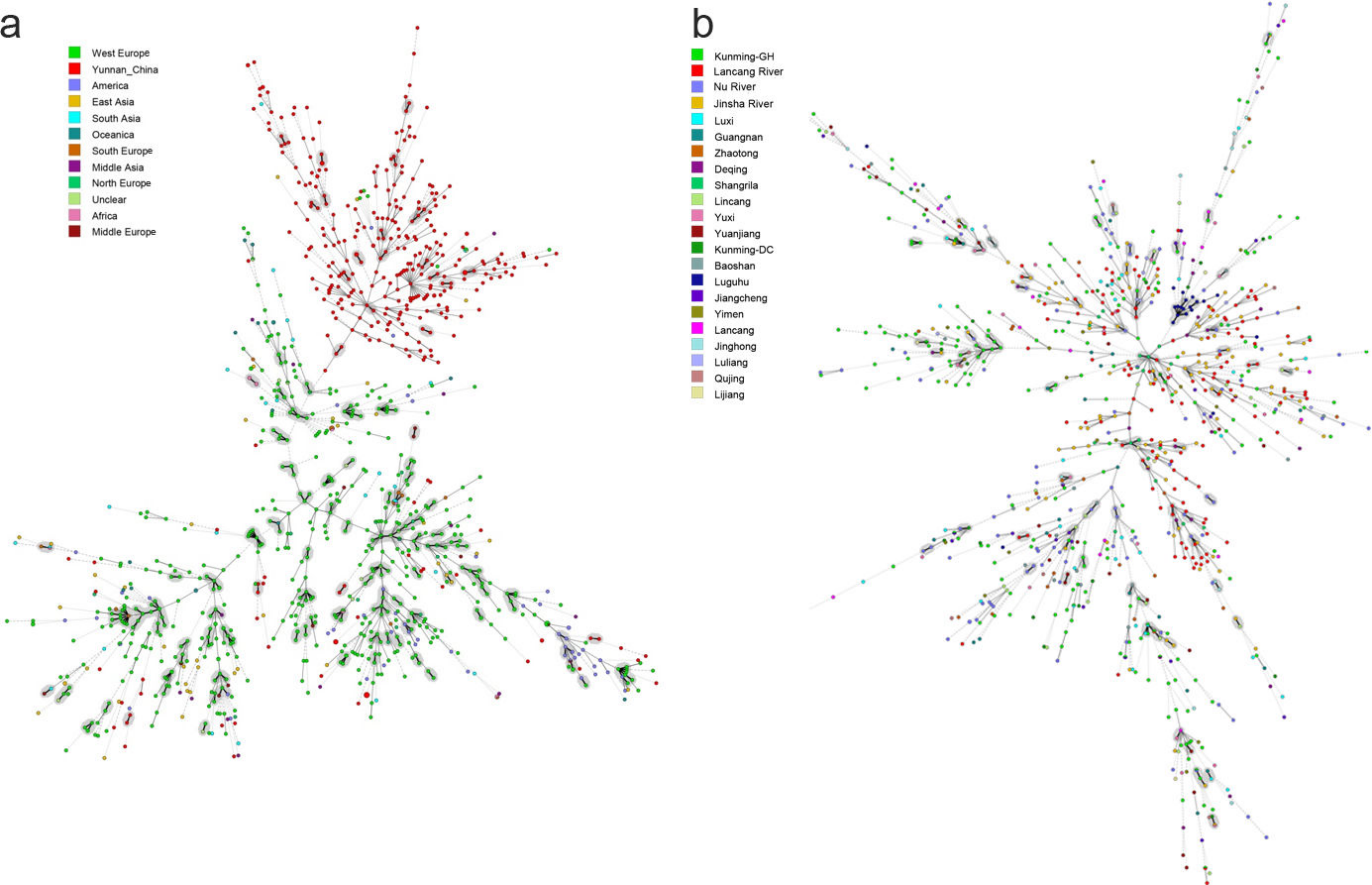
Minimum spanning tree (MST) showing the genotypic relationship among different geographical populations from the world (a) and Yunnan province (b). Each circle corresponds to a unique genotype, and the size of the circle proportionally represents the number of isolates with that genotype. Connecting lines correspond to the number of differences between the genotypes. Short bold line, one difference; black line, two differences; long gray line, three differences; dotted line, four or more differences.

STRUCTURE analyses identified two distinct genetic clusters in the TPR population of *A. fumigatus* (Fig. 3a). The existence of gene flow among geographic populations was supported by cross-distributions of alleles and genotypes in both genetic clusters. However, the composition of genetic elements in the populations from Nu River was distinguished from those other populations. Specifically, the genetic element represented by the color black was the major component among local populations from Nu River. In contrast, that of populations from the other two rivers showed the reverse pattern (Fig. 3c). DAPC further supported the uniqueness of populations along Nu River, that most of the isolates from Jinsha River and Lancang River were clustered together, while those from Nu River mainly were clustered apart from them (Fig. 4a). Meanwhile, consistent with the binary genetic clustering of the *A. fumigatus* isolates we analyzed, phylogenetic analysis results show that the 19 local populations were divided into two clades, the populations from Jinsha River and Lancang River clustered together. In contrast, the populations of Nu River formed a separate clade (Fig. 5). The above results indicated that geographic isolation occurred along the Nu River in the TPR region. However, when combining strains from other geographical populations of Yunnan province analyzed in our previous study into analyses, *A. fumigatus* strains did not form unique clades based on their geographical origin, indicating extensive mixing among *A. fumigatus* populations in Yunnan province (Fig. 6a).

Despite very different compositions of genetic elements among the river-affiliated populations in the TPR region, evidence for recombination was found in all samples. Specifically, in the total sample that includes all 19 local populations, none of the STR loci comparisons showed phylogenetic compatibility (PrC = 0, *P* = 1), consistent with recombination throughout the sites we have tested in the genome. However, the null hypothesis of random mating and recombination in the total sample was rejected (*P* < 0.001).

### Relationship between from the population in the Three Parallel Rivers region and the global populations

We further assessed the relationships between the TPR *A. fumigatus* isolates and those from other countries (regions) deposited in AfumID (https://afumid.shinyapps.io/afumID/). Here, a total of 1,108 *A*. *fumigatus* strains were used. All samples were divided into 11 populations according to their geographical origins (9, 32). Based on nine microsatellite loci, 336 alleles were found, and 251 alleles were shared between the TPR samples and other geographic populations. Of the remaining 85 alleles, 18 and 67 alleles were unique to the TPR populations and the global samples, respectively (Table 5). Similar to our previous studies (9, 32), the #4 private allele at locus 4C appeared with a high frequency (0.78) in the TPR region. Furthermore, a total of 1,009 multilocus genotypes were found from the 1108 *A*. *fumigatus* strains, and no genotype was shared between the TPR and the global samples. Molecular variation analysis showed that 91% of the genetic variation was found within populations and 9% among the global geographic populations (PhiPT = 0.061, *P* =0.001) (Table 3). Pairwise population results showed that among the 66 population pairs, 53 pairs showed statistically significant differentiation (*P* < 0.05), and the populations from TPR were significantly different from all other geographic populations (*P* = 0.001). Among them, the populations with the highest and lowest degrees of genetic differentiation from the TPR population were those from Africa (PhiPT = 0.215, *P* = 0.001) and East Asia (PhiPT = 0.112, *P* = 0.001), respectively (Table S5). The pattern of genetic differentiation was further supported by PCoA results, which showed that the TPR populations were statistically different from other populations, with the African population being the most different from them (Fig. 4b). STRUCTURE analyses identified two distinct genetic clusters among the global samples (Fig. 3b and d). Our samples from Yunnan were relatively distinct from the two global major clades of *A. fumigatus*, commonly referred as populations A and B (14). However, evidence for genetic exchanges were found among the three genetic populations (Fig. 3e and f). Furthermore, PCoA, DAPC (Fig. 4c), and MST (Fig. 6b) analyses with the complete global dataset all showed that the Yunnan population was significantly different from other populations of the world. The first two coordinates of PCoA (PC1+PC2) in combination explained 68.53% of the total observed genetic variation (Fig. 4c).

**TABLE 5:** Similarities and differences in alleles between Yunnan populations of *A. fumigatus* and those from other parts of the world

Locus	No. of alleles in all 12 populations	No. of alleles in Yunnan	Private alleles in Yunnan	Frequency of private alleles
STRAF2A	27	16	8	0.0006
STRAF2B	29	19	31	0.003
STRAF2C	29	22	7, 30 and 36	0.021, 0.003 and 0.003
STRAF3A	83	48	19	0.003
STRAF3B	36	29	44, 49 and 52	0.003, 0.003 and 0.003
STRAF3C	51	41	35, 37, 57 and 64	0.012, 0.003, 0.006 and 0.003
STRAF4A	27	18	21 and 51	0.015 and 0.003
STRAF4B	25	12	17 and 18	0.003 and 0.003
STRAF4C	29	10	4	0.78
Total	336	215	18	

### Prevalence of azole resistance and insertional mutation

Azole susceptibility of 331 representative isolates showed that the MICs of ITR and VOR ranged from 0.125 to ≥ 16 µg/ml (MIC_50_ = 0.5 μg/mL, MIC_90_ = 2.0 μg/mL) and 0.25 to ≥ 8 µg/ml (MIC_50_ = 1.0 μg/mL, MIC_90_ = 2.0 μg/mL), respectively. Among the 331 isolates, 6.95% (23/331) were resistant to one or both clinical triazoles ITR and/or VOR. Specifically, 6.34% (21/331), 3.63% (12/331), and 3.02% (10/331) were resistant to ITR (MIC ≥ 4 mg/L), VOR (MIC ≥ 4 mg/L), and both ITR and VOR, respectively (Table 6). Eleven out of the 19 local populations from the TPR region had ARAF isolates, with seven populations having isolates resistant to both ITR and VOR. The geographical population with the highest frequency of azole resistance was NJ3 at 25% (3/12), followed by JSJ4 at 15% (3/20), and NJ5 at 14.29% (3/21). Of the six populations from along the Nu River, five harbored ARAF with a combined frequency of 11.34% (18/97). In comparison, ARAFs were found in four out of seven populations from the Lancang River basin with an overall frequency of 5.26% (7/133), and in two of six populations from the Jinsha River basin with a frequency of 4.95% (5/101).

**TABLE 6:** Prevalence of azole resistance among 331 *A*. *fumigatus* isolates in 19 local populations from the Three Parallel Rivers region

Sampling site	No. of isolates	No. of isolates resistant to ITR or VOR (MIC ≥ 4 µg/mL)	No. of ITR-resistant isolates	No. of VOR-resistant isolates	No. of cross-resistant isolates
JSJ1	18	2 (11.1%)	1 (5.56%)	1 (5.56%）	0
JSJ2	22	0	0	0	0
JSJ3	18	0	0	0	0
JSJ4	20	3 (15%)	3 (15%)	2 (10%)	2 (10%)
JSJ5	11	0	0	0	0
JSJ6	12	0	0	0	0
LCJ1	21	0	0	0	0
LCJ2	12	0	0	0	0
LCJ3	19	1 (5.26%)	1 (5.26%)	0	0
LCJ4	22	0	0	0	0
LCJ5	16	1 (6.25%)	1 (6.25%)	0	0
LCJ6	21	2 (9.52%)	2 (9.52%)	0	0
LCJ7	22	3 (13.64%)	3 (13.63%)	1 (4.55%)	1 (4.55%)
NJ1	19	2 (10.53%)	2 (10.53%)	2 (10.53%)	2 (10.53%)
NJ2	13	1 (7.69%)	1 (7.69%)	0	0
NJ3	12	3 (25%)	3 (25%)	3 (25%)	3 (25%)
NJ4	13	0	0	0	0
NJ5	21	3 (14.29%)	3 (14.29%)	1 (4.76%)	1 (4.76%)
NJ6	19	2 (10.53%)	1 (5.26%)	2 (10.53%)	1 (5.26%)
Total	331	23 (6.95%)	21 (6.34%)	12 (3.63%)	10 (3.02%)

We obtained the *cyp51A* protein-coding DNA sequence and the promoter sequence for 273 of the 331 isolates, including the 18 of the 23 triazole-resistant ones. The remaining 58 isolates including five triazole-resistant ones either failed the PCR amplification or didn’t yield any clear sequences using the above primers. Table S6 showed the distribution of mutations and insertions at the *cyp51A* gene among the 273 strains with different MICs. The mutations can be broadly grouped into the following two categories. The first group included the following five mutations (F46Y, V172M, T248N, E255D, and K427E) and they are found in almost all the TPR isolates, regardless of their susceptibilities to triazoles. The second group included distinct but diverse mutations found only among the triazole-resistant isolates, including L98H and S297T. Strains with the TR34 insertion all had MIC ≥ 4 mg/mL and all are cross-resistant to both triazoles (Table S6).

## DISCUSSION

### Extensive genetic uniqueness across TPR

In this study, we obtained soil isolates of *A. fumigatus* and used a panel of nine STR markers to identify the genotypes of these isolates and to analyze the genetic diversity within and relationships among populations of this species. STR markers (also called microsatellite markers and simple sequence repeats SSR) are among the most frequently used genetic markers for population genetic and epidemiological studies of a variety of organisms, including animals, plants, and eukaryotic microorganisms such as fungi. In *A. fumigatus*, the nine STR markers have shown to be highly reproducible and highly polymorphic and have helped revealing a diversity of local to global population genetic patterns, including providing evidence for both local clonal expansion and long-distance dispersals (3, 5, 7, 9, 32). However, though highly polymorphic in our samples, these nine markers represent only a very small part of the *A. fumigatus* genome. Indeed, genetic variations among isolates in most of the genome are not analyzed here. Additional markers, including single nucleotide polymorphisms at the whole-genome level, will likely reveal more polymorphisms among our strains and populations than what we found here, as shown in the human pathogenic *Cryptococcus* (40).

In our previous two studies (9, 32), we found widespread genetic uniqueness and extensive genetic diversity of *A. fumigatus* populations from greenhouses and the outdoor environments in Yunnan. For instance, at the same nine STR loci, the number of private alleles and unique genotypes from greenhouses were 40 and 193, accounting for 19.2% (40/208) of the total number of alleles and 97.0% (193/199) of the total number of genotypes, respectively, while the occurrence of those private genetic elements from the outdoor populations in Yunnan were a little lower, at 16.2% (private alleles) and 94.1% (unique genotypes), respectively (9, 32). In this study, a high-level genetic diversity was also found among the 358 *A*. *fumigatus* strains in the TPR populations. For example, we found a total of 215 alleles and 332 genotypes, and most local populations (*n* = 16) had private alleles, accounting for 19.07% (41/215) of the total number of alleles, and the unique genotypes accounting for 98.8% (328/332) of the total number of genotypes. The proportions of private alleles and genotypes are similar to those of *A. fumigatus* populations from Yunnan’s greenhouses (32), but higher than those of *A. fumigatus* populations from various outdoor environments across Yunnan (9). Furthermore, 18 unique alleles were found when comparing our TPR strains and those from AfumID globally, and no shared genotype was found. And, the TPR populations had 14 unique alleles and 317 unique genotypes when compared to those from populations from other areas in Yunnan province. Both PCoA and DAPC results showed that the strains from TPR were clustered differently from most other geographic populations from AfumID. Though PCoA axes in our samples only explained ~70% of the variance, this value was comparable to other studies of *A. fumigatus* or to studies of other organisms such as plants and animals in the same geographic area. In our previous study concerning the genetic diversity of *A. fumigatus* in greenhouses, the first two principal coordinate (PC1 and PC2) in combination explained 76.63% of the total variation (32). Furthermore, genotypic variations from different genomic regions of the sub/alpine herb *Koenigia forrestii* populations in the same geographic area (Himalaya–Hengduan Mountains) contributed about 49.7–55.6% to total variation (41). Similarly, other studies on plants and animals in this geographic region indicated comparable or lower amounts of contributions by the two major principal components to the total genetic variance: e.g., 47.32% was found in the herb *Roscoea humeana* (42) and 35.1% in *Thitarodes moths* (43). Indeed, random sampling effects often show significant influences on observed genetic variations.

Large rivers and high mountains have been identified as gene flow barriers for river-associated, subalpine/alpine plant and animal species, respectively (44, 45). Numerous geographical barriers, such as lofty ridges and deep valleys in TPR region, have been found to greatly restrict gene flow and led to relatively independent evolution among isolated mountain blocks (46, 47). For example, angiosperms in the higher elevational belts in the Eastern Himalaya region, such as the north Hengduan Mountains subregion and the Sanjiang Valley subregion, displayed phylogenetic clustering, suggesting environmental filtering and rapid speciation of many species (44). Interestingly, the ectomycorrhizal basidiomycete mushroom *Tricholoma matsutake* showed a population structure significantly influenced by the mountainous landscape (succession of mountain summits and valleys) in the Eastern Himalaya (48), with watersheds separated by treeless ridge tops in northwest region of Yunnan province containing genetically distinct populations of Matsutake, each of which contributing novel genetic variation to a larger metapopulation. A similar phenomenon was found in the hypogeal fungus *Tuber indicum* species complex: more than 80% (63/77) of the haplotypes were restricted to certain geographic populations in the Hengduan Mountains region in Southwest China (49). In this study, we provided evidence for genetic uniqueness across TPR of the airborne human-pathogenic species *A. fumigatus*, with the high mountains contributing significantly to the observed differentiation among the local TPR populations.

Interestingly, the loci STRAf3A and STRAf3C having the highest number of alleles in the TPR region also had the highest number of alleles in *A. fumigatus* populations from greenhouses and the other outdoor environments in Yunnan (9, 32). Among all the alleles, allele #4 at locus 4C, which was absent in most other geographic populations except in Cameroon, Africa in the global sample (50), was found in very high frequency among 233 isolates from greenhouses in Jinning (42.06%) and 452 isolates from 19 geographic locations across Yunnan (55.30%) (9, 32). We hypothesized that the high frequency of allele 4 at locus 4C might be common in Yunnan *A. fumigatus* populations. Our results here is consistent with this hypothesis: allele #4 at locus 4C was at a very high frequency (80.7%) among the 358 *A*. *fumigatus* strains from the TPR populations. In the Cameroon population, 26.4% (14/53) isolates were found to harbor the same allele (50). The Cameroon populations were obtained from lower elevations than our samples here. At present, the reasons for the shared alleles between these two geographically discrete populations are not known. However, there are three possibilities. In the first, the climate of both Yunnan and Cameroon are divided into rainy and dry seasons each year, which could have contributed to similar accumulation of locally adapted alleles within and among local populations in Yunnan and Cameroon. In the second, there might have been a recent gene flow of the species caused by personnel exchanges including assistance from Yunnan with Cameroon in the construction of hydropower stations, highways, and rubber processing plants, as well as trades of coffee, sugar, and minerals, leading to long distance spread of this specific allele. In the third, the two populations might have been established by the same founder population with allele #4 representing a signature of the founder event.

### Geographically structured genetic differentiations

Even though we found evidence of allele and genotype sharing among the 19 populations from TPR, more than two-thirds (62.33%, 110/171) of our pairwise comparisons among local TPR populations showed significant genetic differentiation (*P* < 0.05) (Table 1). In addition, the overall genetic differentiation among the TPR populations was also significant (PhiPT = 0.040, *P* = 0.001), comparable to our previously analyzed 19 geographic locations across Yunnan (PhiPT = 0.044, *P* = 0.001) (36). However, these results were very different from those from greenhouses in Jinning, Yunnan which showed limited differentiations (PhiPT = 0.019), and from Auckland, New Zealand, where a general lack of genetic differentiation among the six local populations was found (8). Our study also found that river affiliation and altitude differences contributed low but significantly to the overall genetic variation. Melanin in their cell wall and hydrophobicity of the conidia surface enable *A. fumigatus* to be a ubiquitous pathogen with high dispersibility (51), contributing to local and long-distance dispersals and obscuring genetic differentiations among certain geographical and/or ecological populations across national boundaries (52, 53). However, we found a significant positive correlation between altitudes and genetic distances among the local populations from TPR region, while those of the geographical, longitudinal, and latitudinal distances showed a weak and insignificant positive correlation with the genetic distances. The higher the altitude, the greater the genetic distance, suggesting that altitude forced more than other geographical parameters to restrict gene flow among *A. fumigatus* populations across the mountainous area. Feng et al. reported three individual geographic groups of the gourmet mushroom *Tuber indicum* separated by rivers Lancang and Jinsha from TPR, implying that rivers had acted as barriers for gene flow among the species complex (49). Our study also suggested that local geographical barriers (e.g., high ridges and drainage isolation) could block airborne dispersal of spores in *A. fumigatus* and contribute to shape the spatial genetic structure of this species in TRP areas. Moreover, soil texture characteristics of terrace lands varied greatly with altitudes and slope steepness, which affected the distribution of different genotypes.

The generally deep and linear corridors along rivers in TPR may have enforced not only dispersal of genetic elements along altitudinal gradients but also along river drainages. This hypothesis is supported by the high population differentiation documented by Nu River in this region. Interestingly, all the populations from Nu River were significantly different from populations from Jinsha River and Lancang River. The DAPC, PCoA, structure and phylogenetic analysis all showed differences between the *A. fumigatus* populations from Nu River and other two rivers. Furthermore, Nu River populations had the highest genetic diversity and the largest number of private alleles, but the lowest genetic differentiation level. Frequent gene flow may have contributed to maintaining low genetic differentiation and high genetic diversity in Nu River populations. Lastly, the frequency of triazole resistance of *A. fumigatus* in the Nu River population was the highest, followed by the Jinsha River and Lancang River populations. Nu River is located westernmost in TPR area with the lowest altitude among the three main rivers, it is sandwiched between the Biluo Snow Mountain and the Gaoligong Mountain, which are parts of the Hengduan Mountains with an altitude of 4,500 m and 4,000 m, respectively. In addition, the road along the Nu River is the only way for local residents in the river valley to connect with the outside world, thus these areas with high genetic diversity were likely more influenced by anthropogenic factors, with sampling sites close to villages and towns and relatively more frequent human activities, which can promote the gene flow in *A. fumigatus* (8). Indeed, both shared genotypes and unique genetic elements were found in these areas. Moreover, the frequent recombination of *A. fumigatus* we found here can generate genetic variation and may allow faster adaptation to environmental changes, including the spread of alleles conferring triazole resistance along Nu River. Therefore, in addition to dispersal pattern driven by geographical parameters, human-aid local adaption and recombination might have impacted the evolution of *A. fumigatus* in the TPR area. Previous studies suggested the existence of passive dispersal of *A. fumigatus* conidia by soil bacteria such as *Paenibacillus vortex* (54), and other means like rodents, insects, and worms. Since areas with low altitude could foster the reproduction of these organisms, it might be possible to contribute to the observed extensive genetic diversity there.

### Prevalence and putative mechanisms of ARAF in the TPR region

Generally, during prolonged drug exposure, resistant mutations in the *cyp51A* gene causing amino acid substitutions in lanosterol 14α-demethylase involved in the ergosterol biosynthesis pathway could be selected and reduce the overall triazole susceptibility at the population level (39, 55, 56). Up to now, single or pan-azole resistant mutations in *A. fumigatus* have been identified at the *cyp51A* gene, with or without a tandem repeat (TR) insertion within the upstream regulatory region of *cyp51A*, as well as a broad spectrum of triazole susceptibilities in *A. fumigatus* populations around the world (57, 58).

Previous studies before 2021 reported the prevalence of ARAF from various environments in parts of China outside of Yunnan province, with the frequency of ARAF ranging from 1.4% to 10.2% (59 - 61). However, one of our studies in 2021 using strains from nine greenhouses located in Jinning near Kunming, Yunnan province, revealed the highest prevalence of ARAF (79.0%) so far reported in the literature across globe (32). Another study reported 15.89% (58/365) of ARAF from 19 geographic locations across Yunnan and found that the sediments of Dianchi Lake had the highest frequency of ARAF (42.9%, 9/21) among the four ecological niches (agricultural fields, orchards, board-leaved forests, and sediments) (9). Together, these results suggested that agricultural and environmental fungicide usages were potential forces to drive drug resistance in *A. fumigatus* in the non-clinical environments.

Here, we found ARAF strains in 11 of the 19 populations from TPR region. Of the total 331 tested strains, 6.95% (23/331) were resistant to ITR and/or VOR, with 3.92% (10/331) being cross-resistant to both ITR and VOR. The prevalence of ARAF in this study was significantly lower than in our previous two studies in Yunnan. The mountainous terrains of this region are extremely unfavorable to agricultural activities, and the local supplies of vegetables and grains mainly rely on imports from surrounding areas. Consequently, the use of fungicides is relatively uncommon, which is consistent with the lower level of ARAF compared with our previous two studies in Yunnan. However, the prevalence of ARAF in the TRP region was still higher than the strains from hospital gardens, city parks, and farmlands outside Yunnan (1.4%) and that from greenhouses in Zhejiang province (4.1%) (60, 61). It should be noted that the sampling sites in our previous two studies were mainly agricultural fields. Although the local government strongly advocated green agriculture and had strict guidelines for the application of pesticides and fungicides, Yunnan still uses large amounts of triazole fungicides for agricultural production (62, 63). Frequent use of triazole fungicides in agriculture can lead to ARAF in the environment (64 - 67). Therefore, it is not surprising to find a high frequency of ARAF in the agricultural environment of Yunnan, while in this study, all the sampling sites where ARAF were found were non-agricultural fields. There are two possible reasons for ARAF in the TPR regions. Firstly, ARAF can spread from agricultural fields to non-agricultural soils under the influence of human activities, wind, runoff water, and other factors (50). Secondly, the fungicide residues may be carried over by rainwater from agricultural fields into non-agricultural fields and then the residues acted as a selective force for the emergence of ARAF in non-agricultural fields (68).

Among the 331 isolates from the TPR region, 21 and 12 were resistant to ITR and VOR (MIC ≥ 4.0 μg/mL), respectively. Among them, 12 of 21 ITR-resistant isolates had MIC ≥ 16.0 µg/mL, none of 12 VOR-resistant isolates had MIC ≥ 16.0 µg/mL, and the MIC value distributions between ITR and VOR were also different. Further analysis showed that 10 of the 23 triazole-resistant strains were cross-resistant to both ITR and VOR while the rest were resistant to either ITR or VOR, the proportion of cross-resistant isolates was similar to what was found in our previous two studies (9, 32). Together, these results suggest that care must be taken in selecting appropriate drugs for clinical treatment of aspergillosis caused by *A. fumigatus* in Yunnan.

Among the diverse mutations found among our isolates at the *cyp51A* locus, five were broadly distributed in our TPR samples with or without triazole resistance. Whether these mutations are ubiquitous in the remote regions from Eastern Himalaya needs further study. Furthermore, diverse patterns of other mutations occurred in resistant strains, indicating potentially unique selection for azole-resistant strains in this region. Interestingly, the TR34/L98H mutation was the most common mutation associated with triazole (cross-)resistance in environmental samples from Yunnan province, similar to those reported from other parts of China (69, 70), as well as to many other countries around the world (14, 71).

We note that our analyzed samples across the 19 sites were collected at a single time point, during 4 days in early January 2020. While this short period of sampling helps reduce the influence of potential confounding temporal factor in our analyses of geographic factors on population genetic variations, it’s also possible that obtained samples may not fully represent the genetic variations of *A. fumigatus* populations at these sites. Specifically, the warm and raining season between June and October may increase the reproduction of *A. fumigatus* in these regions, leading to higher isolation frequencies of *A. fumigatus*. A recent study showed differences in thermal profiles among isolates from different regional populations of *A. fumigatus* (72). In addition, if asexual reproduction predominates the warm and raining season, we may see stronger signals of clonal population structure within many local populations. Further analyses with additional samples at other seasons are needed to determine the representativeness of our current samples for *A. fumigatus* populations in this region and the potential effects of seasons on population genetic structure of *A. fumigatus*.

## SUPPLEMENTAL MATERIAL

Supplemental Tables msphere.00071-23-s0001.docx Tables S1 to S6.

ASM does not own the copyrights to Supplemental Material that may be linked to, or accessed through, an article. The authors have granted ASM a non-exclusive, world-wide license to publish the Supplemental Material files. Please contact the corresponding author directly for reuse.
