# Supplementary material for: Genetic structure and triazole resistance among Aspergillus fumigatus populations from remote and undeveloped regions in Eastern Himalaya
Source: mSphere. 2023 Jun 21;8(4):e00071-23. doi: 10.1128/msphere.00071-23 (PMC10449526; doi:10.1128/msphere.00071-23)
Supplement: Supplemental Tables — Tables S1 to S6. [file msphere.00071-23-s0001.docx]

Supplementary Tables

Table S1. Detailed information of geographical distribution of sampling sites.

| Three Parallel Rivers | Sampling site | Latitude | Longitude | Altitude | Ecological niche |
| --- | --- | --- | --- | --- | --- |
|  |  |  |  |  |  |
| Jinsha  River | JSJ1 | 26.9344 | 99.9581 | 1830 | Roadside |
|  | JSJ2 | 27.2217 | 99.7436 | 1860 | Forest soil |
|  | JSJ3 | 27.4939 | 99.5672 | 1980 | Forest soil |
|  | JSJ4 | 27.7775 | 99.4308 | 1950 | Hillside |
|  | JSJ5 | 28.0667 | 99.4089 | 2470 | Hillside |
|  | JSJ6 | 28.2672 | 99.2914 | 1680 | Roadside |
| Lancang River | LCJ1 | 28.2228 | 98.8569 | 1940 | Roadside |
|  | LCJ2 | 27.9525 | 98.9119 | 1950 | Roadside |
|  | LCJ3 | 27.7319 | 99.055 | 1650 | Roadside |
|  | LCJ4 | 27.4622 | 99.0447 | 1790 | Vegetable field |
|  | LCJ5 | 27.1914 | 99.1119 | 1630 | Hillside |
|  | LCJ6 | 27.0494 | 99.1633 | 1670 | Hillside |
|  | LCJ7 | 26.8314 | 99.1772 | 1850 | Hillside |
| Nu River | NJ1 | 26.6744 | 98.895 | 1160 | Village soil |
|  | NJ2 | 26.9389 | 98.8658 | 1220 | Village soil |
|  | NJ3 | 27.2603 | 98.8814 | 1290 | Village soil |
|  | NJ4 | 27.4817 | 98.8297 | 1360 | Hillside |
|  | NJ5 | 27.7497 | 98.6719 | 1450 | Hillside |
|  | NJ6 | 28.0183 | 98.6189 | 1680 | Hillside |

Table S2 Analysis of variance using the mean value of diversity estimates of each river.

|  | Jinsha | Lancang | Nujiang |
| --- | --- | --- | --- |
| Jinsha |  | 0.505 | 0.314 |
| Lancang | 0.07 |  | 0.101 |
| Nujiang | 0.1 | 0.17 |  |

Note: Mean deviation values are shown below diagonal. P values are shown above diagonal.

Table S3. Grouping information and diversity of individual populations.

| Category | Grouping | Local population | Unbiased Diversity(uh) |
| --- | --- | --- | --- |
| Rivers | Jinsha River | JSJ1, JSJ2, JSJ3, JSJ4, JSJ5, JSJ6 | 0.754 |
|  | Lancang River | LCJ1, LCJ2, LCJ3, LCJ4, LCJ5, LCJ6, LCJ7 | 0.705 |
|  | Nu River | NJ1, NJ2, NJ3, NJ4, NJ5, NJ6 | 0.838 |
| Altitudes | A1160-1290 | NJ1, NJ2, NJ3 | 0.803 |
|  | A1360-1450 | NJ4, NJ5 | 0.860 |
|  | A1630-1680 | LCJ3, LCJ5, LCJ6, JSJ6, NJ6 | 0.758 |
|  | A1790-1860 | JSJ1, JSJ2, LCJ4, LCJ7 | 0.717 |
|  | A1940-1980 | JSJ3, JSJ4, LCJ1, LCJ2 | 0.748 |
|  | A2470 | JSJ5 | 0.689 |
| Upstream and Downstream | Lat01 | NJ2, LCJ6, JSJ1 | 0.762 |
|  | Lat02 | NJ3, LCJ5, JSJ2 | 0.74 |
|  | Lat03 | LCJ4, NJ4, JSJ3 | 0.722 |
|  | Lat04 | LCJ3, NJ5, JSJ4 | 0.804 |
|  | Lat05 | LCJ1, NJ6, JSJ5 | 0.792 |

Table S4. Pairwise differentiations among 19 local populations of *A. fumigatus* along the Three Parallel Rivers.

| JSJ1 | JSJ2 | JSJ3 | JSJ4 | JSJ5 | JSJ6 | LCJ1 | LCJ2 | LCJ3 | LCJ4 | LCJ5 | LCJ6 | LCJ7 | NJ1 | NJ2 | NJ3 | NJ4 | NJ5 | NJ6 |  |
| --- | --- | --- | --- | --- | --- | --- | --- | --- | --- | --- | --- | --- | --- | --- | --- | --- | --- | --- | --- |
|  | 0.231 | 0.129 | 0.434 | 0.196 | 0.053 | 0.455 | 0.16 | 0.327 | 0.013 | 0.292 | 0.005 | 0.114 | 0.002 | 0.054 | 0.001 | 0.034 | 0.006 | 0.012 | JSJ1 |
| 0.007 |  | 0.061 | 0.066 | 0.025 | 0.011 | 0.074 | 0.01 | 0.452 | 0.019 | 0.359 | 0.002 | 0.031 | 0.001 | 0.001 | 0.001 | 0.001 | 0.001 | 0.001 | JSJ2 |
| 0.012 | 0.018 |  | 0.038 | 0.097 | 0.008 | 0.446 | 0.353 | 0.348 | 0.076 | 0.112 | 0.001 | 0.001 | 0.001 | 0.001 | 0.001 | 0.003 | 0.001 | 0.001 | JSJ3 |
| 0 | 0.016 | 0.023 |  | 0.019 | 0.025 | 0.26 | 0.156 | 0.149 | 0.003 | 0.465 | 0.003 | 0.288 | 0.001 | 0.01 | 0.001 | 0.038 | 0.002 | 0.021 | JSJ4 |
| 0.014 | 0.031 | 0.028 | 0.039 |  | 0.293 | 0.152 | 0.01 | 0.046 | 0.005 | 0.04 | 0.004 | 0.011 | 0.001 | 0.001 | 0.001 | 0.011 | 0.003 | 0.005 | JSJ5 |
| 0.027 | 0.042 | 0.048 | 0.029 | 0.007 |  | 0.05 | 0.007 | 0.028 | 0.001 | 0.051 | 0.001 | 0.041 | 0.005 | 0.012 | 0.002 | 0.447 | 0.048 | 0.042 | JSJ6 |
| 0 | 0.014 | 0 | 0.006 | 0.015 | 0.021 |  | 0.232 | 0.441 | 0.012 | 0.426 | 0.01 | 0.073 | 0.001 | 0.002 | 0.001 | 0.012 | 0.002 | 0.001 | LCJ1 |
| 0.013 | 0.038 | 0.004 | 0.012 | 0.054 | 0.057 | 0.01 |  | 0.181 | 0.014 | 0.301 | 0.008 | 0.276 | 0.004 | 0.011 | 0.007 | 0.023 | 0.005 | 0.007 | LCJ2 |
| 0.003 | 0 | 0.002 | 0.011 | 0.031 | 0.033 | 0 | 0.011 |  | 0.294 | 0.441 | 0.011 | 0.166 | 0.001 | 0.001 | 0.001 | 0.001 | 0.001 | 0.001 | LCJ3 |
| 0.034 | 0.026 | 0.017 | 0.041 | 0.085 | 0.091 | 0.027 | 0.037 | 0.005 |  | 0.301 | 0.001 | 0.001 | 0.001 | 0.001 | 0.001 | 0.001 | 0.001 | 0.001 | LCJ4 |
| 0.006 | 0.002 | 0.015 | 0 | 0.034 | 0.029 | 0.002 | 0.006 | 0 | 0.003 |  | 0.014 | 0.308 | 0.001 | 0.002 | 0.001 | 0.017 | 0.001 | 0.003 | LCJ5 |
| 0.043 | 0.041 | 0.062 | 0.039 | 0.064 | 0.07 | 0.035 | 0.051 | 0.031 | 0.082 | 0.038 |  | 0.01 | 0.001 | 0.001 | 0.001 | 0.005 | 0.001 | 0.001 | LCJ6 |
| 0.011 | 0.02 | 0.046 | 0.005 | 0.043 | 0.024 | 0.015 | 0.007 | 0.01 | 0.049 | 0.003 | 0.032 |  | 0.002 | 0.027 | 0.001 | 0.059 | 0.001 | 0.004 | LCJ7 |
| 0.041 | 0.08 | 0.086 | 0.051 | 0.092 | 0.041 | 0.058 | 0.045 | 0.072 | 0.117 | 0.071 | 0.077 | 0.036 |  | 0.468 | 0.198 | 0.069 | 0.46 | 0.011 | NJ1 |
| 0.025 | 0.071 | 0.073 | 0.028 | 0.085 | 0.039 | 0.053 | 0.049 | 0.057 | 0.098 | 0.063 | 0.069 | 0.025 | 0 |  | 0.302 | 0.17 | 0.455 | 0.028 | NJ2 |
| 0.064 | 0.118 | 0.101 | 0.06 | 0.111 | 0.057 | 0.074 | 0.056 | 0.097 | 0.141 | 0.089 | 0.064 | 0.049 | 0.011 | 0.006 |  | 0.422 | 0.063 | 0.007 | NJ3 |
| 0.027 | 0.068 | 0.063 | 0.025 | 0.051 | 0.001 | 0.033 | 0.039 | 0.065 | 0.104 | 0.043 | 0.055 | 0.022 | 0.019 | 0.014 | 0.002 |  | 0.169 | 0.045 | NJ4 |
| 0.032 | 0.083 | 0.076 | 0.041 | 0.064 | 0.02 | 0.044 | 0.053 | 0.077 | 0.122 | 0.075 | 0.083 | 0.043 | 0 | 0 | 0.017 | 0.01 |  | 0.077 | NJ5 |
| 0.028 | 0.076 | 0.064 | 0.022 | 0.053 | 0.028 | 0.04 | 0.04 | 0.062 | 0.099 | 0.042 | 0.076 | 0.034 | 0.029 | 0.025 | 0.038 | 0.023 | 0.015 |  | NJ6 |

Note: PhiPT Values below diagonal. Probability, P (rand >= data) based on 999 permutations is shown above diagonal.

Table S5. Pairwise differentiations between *A. fumigatus* populations from 12 geographic regions around the globe.

| Am | SA | EA | MA | Af | SE | ME | NE | WE | Oc | Un | YC |  |
| --- | --- | --- | --- | --- | --- | --- | --- | --- | --- | --- | --- | --- |
|  | 0.001 | 0.001 | 0.001 | 0.001 | 0.003 | 0.025 | 0.025 | 0.001 | 0.001 | 0.004 | 0.001 | Am |
| 0.057 |  | 0.144 | 0.462 | 0.043 | 0.023 | 0.003 | 0.001 | 0.001 | 0.242 | 0.001 | 0.001 | SA |
| 0.047 | 0.006 |  | 0.453 | 0.001 | 0.028 | 0.007 | 0.002 | 0.001 | 0.007 | 0.001 | 0.001 | EA |
| 0.063 | 0.001 | 0.000 |  | 0.010 | 0.116 | 0.017 | 0.001 | 0.005 | 0.014 | 0.010 | 0.001 | MA |
| 0.183 | 0.070 | 0.120 | 0.099 |  | 0.056 | 0.005 | 0.002 | 0.001 | 0.018 | 0.004 | 0.002 | Af |
| 0.048 | 0.038 | 0.025 | 0.021 | 0.144 |  | 0.144 | 0.409 | 0.223 | 0.037 | 0.312 | 0.001 | SE |
| 0.032 | 0.057 | 0.033 | 0.041 | 0.187 | 0.029 |  | 0.034 | 0.043 | 0.001 | 0.024 | 0.001 | ME |
| 0.032 | 0.083 | 0.050 | 0.070 | 0.222 | 0.000 | 0.045 |  | 0.181 | 0.001 | 0.330 | 0.001 | NE |
| 0.031 | 0.033 | 0.024 | 0.030 | 0.128 | 0.005 | 0.021 | 0.009 |  | 0.001 | 0.111 | 0.001 | WE |
| 0.063 | 0.007 | 0.025 | 0.038 | 0.109 | 0.046 | 0.066 | 0.078 | 0.036 |  | 0.006 | 0.001 | Oc |
| 0.049 | 0.062 | 0.049 | 0.048 | 0.185 | 0.008 | 0.057 | 0.006 | 0.009 | 0.072 |  | 0.001 | Un |
| 0.141 | 0.138 | 0.112 | 0.123 | 0.215 | 0.133 | 0.146 | 0.150 | 0.120 | 0.140 | 0.145 |  | YC |

Note: PhiPT Values below diagonal. Probability, P (rand >= data) based on 999 permutations is shown above diagonal. AM, America; SA, South Asia; EA, East Asia; MA, middle Asia; Af, Africa; SE, south Europe; ME, middle Europe; NE, north Europe; WE, west Europe; Oc, Oceanica.

UN, unclear regions; YC, the Three Parallel Rivers, Yunnan, China

Table S6. Distribution of mutations and insertions among 273 strains with different minimum inhibitory concentrations against itraconazole (ITR) and voriconazole (VOR).

| **MIC (μg/mL)** | **ITR** | **Y46F** | **L98H** | **V172M** | **L221V** | **T248N** | **T248K** | **E255D** | **D262A** | **S297T** | **K427E** | **TR34** |
| --- | --- | --- | --- | --- | --- | --- | --- | --- | --- | --- | --- | --- |
| >16 | 10 | 10 | 4 | 10 | 0 | 9 | 8 | 10 | 0 | 1 | 8 | 5 |
| 8 | 0 | 0 | 0 | 0 | 0 | 0 | 0 | 0 | 0 | 0 | 0 | 0 |
| 4 | 7 | 7 | 0 | 7 | 1 | 6 | 1 | 7 | 0 | 0 | 7 | 0 |
| 2 | 30 | 30 | 0 | 30 | 1 | 28 | 2 | 30 | 0 | 0 | 30 | 0 |
| 1 | 59 | 59 | 0 | 59 | 2 | 58 | 0 | 58 | 2 | 0 | 58 | 0 |
| 0.5 | 100 | 100 | 0 | 100 | 0 | 96 | 3 | 99 | 0 | 0 | 100 | 0 |
| 0.25 | 52 | 52 | 1 | 52 | 0 | 48 | 2 | 50 | 3 | 0 | 50 | 0 |
| 0.125 | 15 | 15 | 0 | 15 | 0 | 14 | 1 | 15 | 1 | 0 | 15 | 0 |
|  | **VOR** |  |  |  |  |  |  |  |  |  |  |  |
| >16 | 0 | 0 | 0 | 0 | 0 | 0 | 0 | 0 | 0 | 0 | 0 | 0 |
| 8 | 4 | 4 | 2 | 4 | 0 | 4 | 0 | 4 | 0 | 0 | 4 | 3 |
| 4 | 5 | 5 | 2 | 5 | 0 | 4 | 0 | 3 | 0 | 3 | 3 | 2 |
| 2 | 45 | 45 | 0 | 45 | 0 | 42 | 1 | 43 | 1 | 0 | 43 | 1 |
| 1 | 158 | 158 | 1 | 158 | 4 | 151 | 5 | 157 | 5 | 0 | 157 | 0 |
| 0.5 | 57 | 57 | 0 | 57 | 0 | 54 | 2 | 56 | 1 | 0 | 57 | 0 |
| 0.25 | 4 | 4 | 0 | 4 | 0 | 0 | 1 | 4 | 0 | 0 | 4 | 0 |
| 0.125 | 0 | 0 | 0 | 0 | 0 | 0 | 0 | 0 | 0 | 0 | 0 | 0 |
| ITR&VOR≥2 | 36 | 36 | 4 | 36 | 1 | 35 | 0 | 34 | 0 | 1 | 34 | 5 |
| ITR&VOR≥4 | 8 | 8 | 4 | 8 | 0 | 7 | 0 | 6 | 0 | 1 | 6 | 5 |
